# The feasibility of disposable video‐bronchoscopes, Ambu aScope3, for exploration of the common bile duct and extraction of stones

**DOI:** 10.1002/deo2.67

**Published:** 2021-11-21

**Authors:** Sujala N. R. Kalipershad, Daren A. Subar, Ravindra S. Date

**Affiliations:** ^1^ Department of Surgery Lancashire Teaching Hospitals NHS Foundation Trust Lancashire UK; ^2^ Department of Surgery East Lancashire Teaching Hospitals NHS Trust Lancashire UK; ^3^ The University of Manchester Manchester Academic Health Science Centre Manchester UK

**Keywords:** Ambu aScope, common bile duct exploration, common bile duct stones, choledochoscopy, stone extraction

## Abstract

**Introduction:**

The aim of this study was to assess and evaluate the feasibility of using the Ambu aScope3 (aScope) for common bile duct (CBD) explorations, stone detection and extraction, as well as to assess feasibility of its use through the laparoscopic ports in a low‐volume centre.

**Methods:**

This is a dual centre prospective study, conducted between February 2015 and August 2019, of patients undergoing laparoscopic cholecystectomy and common bile duct exploration. Ethical approval was obtained from the North West ‐ Greater Manchester South Research Ethics Committee. All patients were counselled on the use of the aScope in clinic, prior to surgery. The Primary endpoints were the ability of the aScope to identify CBD stones, perform a cholangiogram through the available channel, retrieve the stones using a Dormia basket and to visualise second generation biliary radicles satisfactorily. The secondary endpoint was the use of the aScope, via a laparoscopic port without a gas leak. The data collected included patient demographics, need for a CBD exploration, intraoperative confirmation of CBD stones and their safe extraction using an aScope.

**Results:**

A total of nine patients were recruited. The aScope provided satisfactory views in eight of nine patients and enabled the safe extraction of CBD stones in six of nine cases. One patient had a bile leak, and another had a transected CBD prior to the use of the aScope.

**Conclusion:**

We found that the aScope is a safe, feasible alternative to a choledochoscope, and in a low‐volume centre, it provides a financially viable option.

## INTRODUCTION

The most common intervention for common bile duct (CBD) stones is removal by endoscopic retrograde cholangio‐pancreatography (ERCP). Some of these stones may be difficult to remove endoscopically due to technical reasons, for example previous gastric bypass surgery. For patients who cannot have their stones removed by ERCP, surgical exploration of their CBD is an alternate treatment. Choledochoscopy for CBD exploration is a relatively uncommon procedure compared to other forms of endoscopic procedures for example, gastroscopy or colonoscopy. In these instances, a choledochoscope is used mainly to identify and extract stones from the CBD, and detailed visualisation of mucosa is not necessary during the procedure.

In a small volume centre, such as ours, with a limited number of cases per year, investment in video‐choledochoscopes may not be cost effective, and most centres continue to use direct viewing choledochscopes, where available. A disposable, flexible fibreoptic video‐bronchoscope Ambu aScope (Ambu UK Ltd, Cambridgeshire) is widely used by anaesthetists to visualise the trachea and guide the endotracheal tube during difficult intubations. The aScope has been used successfully in other centres, both within the UK^1^ and internationally,^2^ for CBD exploration and ductal stone extraction. Our aim was to conduct a feasibility study to assess and evaluate the use of the aScope, instead of a choledochoscope, for the visualisation of the bile duct and the successful extraction of CBD stones. This would provide a safe, cost‐effective alternative, in low volume institutes or those that do not have a choledochoscope.

## METHODOLOGY

**FIGURE 1:**
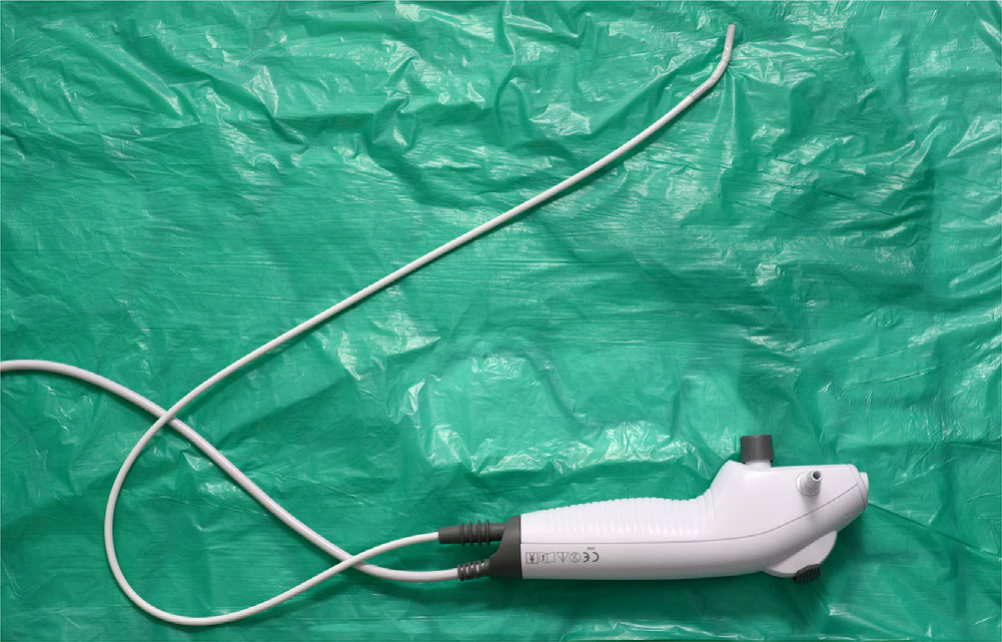
Ambu aScope 3

**FIGURE 2:**
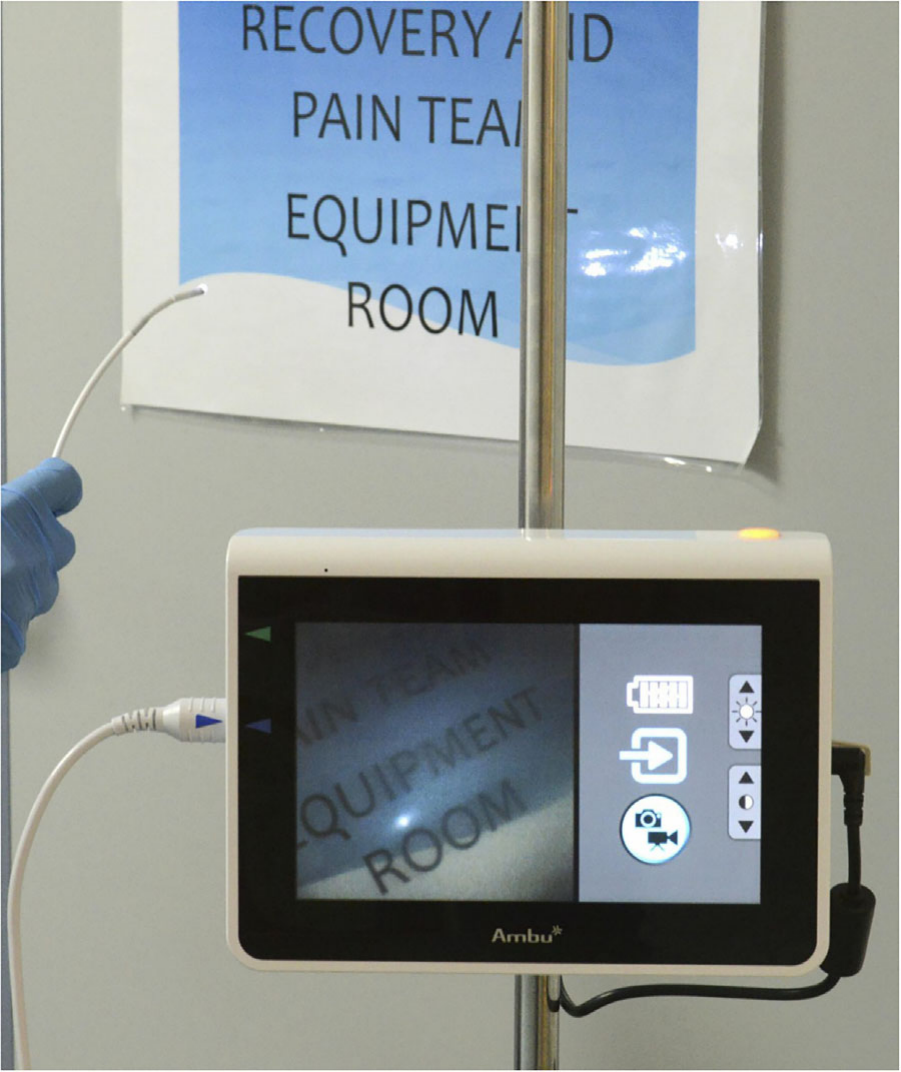
A view monitor to be used with the aScope

This was a prospective study, designed to assess the feasibility of the aScope in the treatment of CBD stones. Ethics approval was attained from the North West ‐ Greater Manchester South Research Ethics Committee 16/NW/0431, as the aScope is still currently unlicensed for use in CBD exploration, in the UK. The study period was from February 2015 to August 2019. The data were recorded for patient demographics, indication for procedure, if ductal stones were suspected or confirmed, views using the aScope, successful extraction of stones, complications and difficulties encountered with the aScope. Ambu aScope3 was used for the procedures (Figure 1) along with its view monitor (Figure 2). aScope3 has insertion cord diameter of 5.0 mm, working channel diameter of 2.2 mm and bending angle 150°/130°.

The inclusion criteria were consenting patients requiring CBD exploration following confirmation or high suspicion of a CBD stone. Patients were excluded from the study if they were unfit for surgery and those who declined to consent. All eligible patients were given an information leaflet with sufficient time for perusal and the opportunity to ask questions, prior to written consent being obtained from willing participants as per good clinical practice guidelines.^3^

## STUDY DESIGN

In the absence of any previous experience with this scope as a choledochoscope, a cautious approach was adopted. The aScope would be used in conjunction with existing choledochoscopes for three cases. If it could be used as effectively and safely as current choledochoscopes, three more patients would be recruited. The cases will be recruited in the batches of three pending the results of previous batch. First 10 cases will be done jointly by two consultants.

A Hepatopancreaticobiliary (HPB) surgeon will be involved and kept on stand‐by during the study. In the event of any major mishap like injury to the bile duct, he will be contacted. Minor problems will be resolved locally. In the event of a major complication, the study will be stopped. An HPB surgeon at a tertiary centre recruited and carried out four procedures, due to small numbers at the initial study site.

The primary endpoint was the ability of the aScope to identify and retrieve all the stones in the CBD, using a Dormia basket through the channel of the aScope. The secondary endpoint was the ability to visualise second generation biliary radicles satisfactorily and also to use the aScope intraoperatively, via a laparoscopic port without a gas leak.

## PROCEDURE

There was no difference in the procedure when compared with conventional choledochoscope. The cystic artery was double clipped, ligated and divided close to the gallbladder. An intraoperative cholangiogram was performed through the cystic duct to confirm the presence of stones in CBD and then double clipped, ligated and divided. The gallbladder was kept in situ to maintain traction on fundus and to give a good view of the CBD. The CBD was clearly defined. Two stay sutures were not taken on CBD as in open operations. A vertical incision was made on the CBD, just superior to the duodenum using laparoscopic scissors. The CBD was opened, and the ascope was inserted through the epigastric port (12 mm Excel port) for exploration of bile duct. Continuous irrigation with saline is done to maintain the view.

A Dormia basket was used for retrieval of the stones through the channel of the scope. A closed basket was passed beyond the stone. The basket was opened and withdrawn slowly to encompass the stone. Once fully encompassed, the basket was tightened around the stone, and the aScope was withdrawn slowly along with the basket and stone, through the choledocotomy. All the stones were retrieved in a plastic bag along with gallbladder at the end of operation. Clearance of ducts up to second generation was confirmed by the aScope. The choledchotomy was closed using interrupted 3/0 PDS suture. A drain was routinely used to monitor for a bile leak from the suture line.

## RESULTS

**FIGURE 3:**
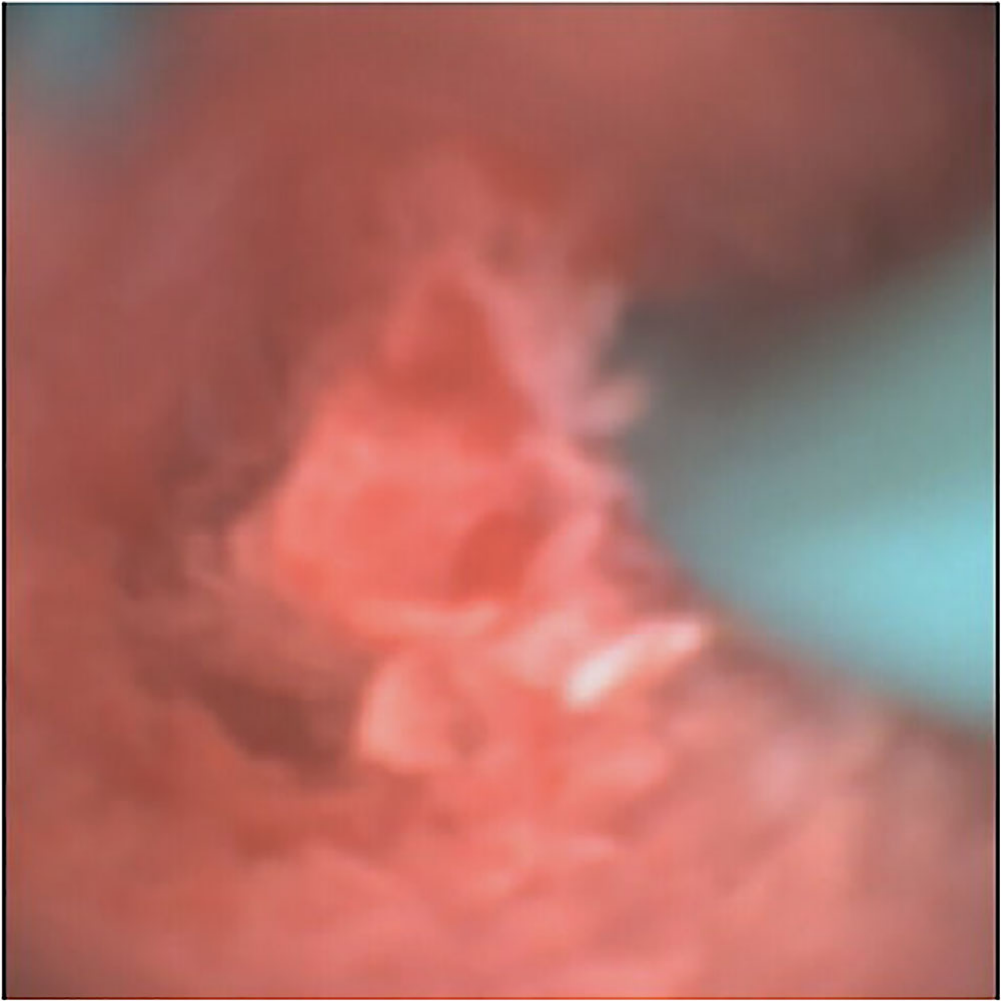
Lower end of the common bile duct with previously inserted pig tail catheter

**FIGURE 4:**
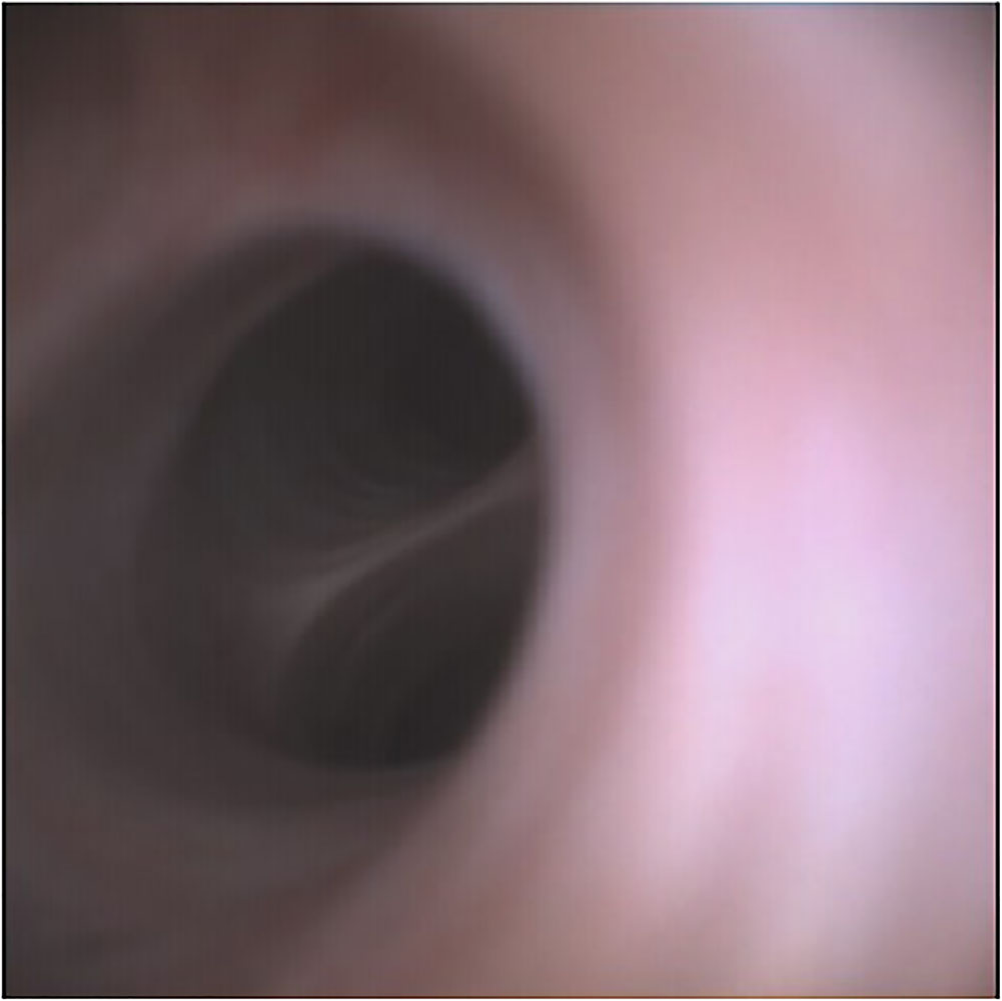
Right and left hepatic duct opening

Nine patients were recruited to the study (Table 1), of which 88.9% had confirmed CBD stones, and 11.1% had a suspected stone. The male to female ratio was 4:5, and the median age was 53. The aScope provided acceptable views of the CBD in 88.9% of cases (Figures 3 and 4). It was successfully able to retrieve the stones in 66.6% of cases; in one patient the views were inadequate, in a second patient there were no stones within the CBD, and the third had a transected CBD prior to the use of the aScope. Second generation biliary radicles were not visualised in 44.4% of cases. There were no problems with the use of the Dormia basket and the ability to secure a gas seal. In one case, the patient had Mirrizi's syndrome, and the CBD was transected prior to the use of the aScope, and another patient had a bile leak.

**TABLE 1:** Proforma for aScope study

Patient number	Age	Gender (M/F)	Indication (CBD stones confirmed or suspected)	Acceptable views (Y/N)	Retrieval of all stones with Dormia basket	Visualise second generation biliary radicles	Complications (CO2 leak from port or CBD injury/transection)
1	40	M	Confirmed	Y	Y	Y	N
2	74	M	Confirmed	Y	Y	Y	N
3	34	M	Confirmed	Y	N	N	CBD divided prior to aScope use
4	81	F	Confirmed	Y	Y	Y	N
5	61	F	Confirmed	Y	No stones in CBD	Y	N
6	48	F	Confirmed	Y	Y	N	N
7	66	F	Confirmed	Y	Y	Y	N
8	53	F	Confirmed	N	N	N	N
9	41	M	Suspected	Y	Y	N	Bile leak

Abbreviation: CBD, common bile duct.

## DISCUSSION

To the best of our knowledge, this is the first ethically approved prospective study looking at the feasibility of using an aScope for CBD explorations. This study supports other published reports where aScopes have been used successfully, concluding that it is safe, effective and cost‐effective.^1^, ^2^, ^4^ The drawback of the aScope is that the resolution^5^ is not as good as conventional bronchoscopes used for Bronchoscopies, but the high resolution is not necessary for the identification of CBD stones. Furthermore, heated devices cannot be used through the aScope for therapeutic purposes, but the aScope is adequate to establish a diagnosis of CBD stones and successfully extract the stones.

Being single‐use and relatively inexpensive compared to a purpose‐built video choledochoscope, the aScope is a cost‐effective alternative for institutions doing a small number of cases, as reported in previous studies.^1^, ^2^, ^4^ Choledochoscopy does not require a very high‐resolution images for the detection of stones. We suggest that the aScope could be used for exploration of the CBD as it is similar in design to a choledochoscope. The aScope has the advantage of a small portable video monitor which allows for better visualisation of the duct, enabling the assistant and theatre team to see the procedure. This is opposed to a direct viewing scope which provides a view to the surgeon alone. Furthermore, a conventional direct viewing scope (that does not transfer images to video screen) is difficult to handle during an operation, as it is held with sterile gloved hands, close to an unsterile eye or face mask, thereby increasing the risk of desterilising the scope. The cost of a disposable aScope is considerably less than available video choledochoscopes, and with small number of procedures performed in our unit, we are unable to justify the cost of a choledochoscope. Other disadvantages of conventional choledochoscopes are the disinfection process, service maintenance, the unavailability of the scope when being serviced and an additional cost for transportation if the scope is disinfected at a central site.

Following our study, we feel that it is safe and effective to use an aScope as an alternative to a choledochoscope for diagnostic and therapeutic purposes. We feel that this is a financially feasible alternative to a purpose designed video choledochoscope, which costs in the region of £20000 and has the associated sterilization, service and maintenance costs^4^. This expenditure is difficult to justify in the current healthcare climate and in low‐volume centres where it would take a number of years to recoup the expense. The aScope in comparison has a much lesser cost of approximately £400 and is a single‐use system hence does not require sterilization or maintenance.

The limitation of our study is the small number of cases, and this is mirrored in the literature.^1^, ^2^, ^4^ A larger study in a high‐volume centre may provide more evidence regarding the possible financial benefits of the aScope. The trend of using disposable scopes in other areas of the body has commenced while conducting this study,^6^, ^7^ and it is likely to be applied to CBD exploration as well.

## CONFLICT OF INTEREST

The authors declare that there is no conflict of interest that could be perceived as prejudicing the impartiality of the research reported.

## FUNDING INFORMATION

Ambu UK Ltd, Cambridgeshire
